# Assessing GPT and DeepL for terminology translation in the medical domain: A comparative study on the human phenotype ontology

**DOI:** 10.1186/s12911-025-03075-8

**Published:** 2025-07-01

**Authors:** Richard Noll, Alexandra Berger, Dominik Kieu, Tobias Mueller, Ferdinand O. Bohmann, Angelina Müller, Svea Holtz, Philipp Stoffers, Sebastian Hoehl, Oya Guengoeze, Jan-Niklas Eckardt, Holger Storf, Jannik Schaaf

**Affiliations:** 1 Goethe University Frankfurt, University Medicine Frankfurt, Institute of Medical Informatics, Theodor-Stern-Kai 7, 60590 Frankfurt am Main, Germany; 2 Goethe University Frankfurt, University Hospital Frankfurt, Frankfurt Reference Centre for Rare Diseases, Frankfurt, Germany; 3 Faculty of Health Sciences, University of Applied Sciences, Giessen, Germany; 4 Department of Neurology, Goethe University Frankfurt, University Hospital Frankfurt, Frankfurt, Germany; 5 Goethe University Frankfurt, University Hospital Frankfurt, Institute of General Practice, Frankfurt, Germany; 6 Charité - Universitätsmedizin Berlin, Department of Hepatology and Gastroenterology, Campus Virchow-Klinikum (CVK) and Campus Charité Mitte (CCM), Berlin, Germany; 7 Digital Health Center, Hasso Plattner Institute, University of Potsdam, Potsdam, Germany; 8 Institute of Medical Virology, Goethe University Frankfurt, University Hospital Frankfurt, Frankfurt, Germany; 9 Department of Internal Medicine 1, Goethe University Frankfurt, University Hospital Frankfurt, Frankfurt, Germany; 10 Department of Medical Information Systems and Digitalization, Goethe University Frankfurt, University Hospital Frankfurt, Frankfurt, Germany; 11 Department of Internal Medicine I, University Hospital Carl Gustav Carus, Technical University Dresden, Dresden, Germany; 12 Else Kroener Fresenius Center for Digital Health, Technical University Dresden, Dresden, Germany

**Keywords:** Artificial intelligence, Controlled vocabulary, Translations, GPT

## Abstract

**Background:**

This paper presents a comparative study of two state-of-the-art language models, OpenAI’s GPT and DeepL, in the context of terminology translation within the medical domain.

**Methods:**

This study was conducted on the human phenotype ontology (HPO), which is used in medical research and diagnosis. Medical experts assess the performance of both models on a set of 120 translated HPO terms and their 180 synonyms, employing a 4-point Likert scale (strongly agree = 1, agree = 2, disagree = 3, strongly disagree = 4). An independent reference translation from the HeTOP database was used to validate the quality of the translation.

**Results:**

The average Likert rating for the selected HPO terms was 1.29 for GPT-3.5 and 1.37 for DeepL. The quality of the translations was also found to be satisfactory for multi-word terms with greater ontological depth. The comparison with HeTOP revealed a high degree of similarity between the models’ translations and the reference translations.

**Conclusions:**

Statistical analysis revealed no significant differences in the mean ratings between the two models, indicating their comparable performance in terms of translation quality. The study not only illustrates the potential of machine translation but also shows incomplete coverage of translated medical terminology. This underscores the relevance of this study for cross-lingual medical research. However, the evaluation methods need to be further refined, specific translation issues need to be addressed, and the sample size needs to be increased to allow for more generalizable conclusions.

## Background

Terminology translation is a crucial task in the field of medical informatics, as it allows for the sharing and integration of knowledge across different languages and medical domains. Medical ontologies are formal representations of medical knowledge that enable the semantics of medical concepts and relationships to be expressed in a structured and machine-readable format. They play a vital role in many medical applications, such as clinical decision support and biomedical research [1].

However, the translation of ontologies and their medical terminology is a challenging task, as it requires expertise in both the source and target languages and a deep understanding of domain-specific concepts and relationships. Recently, interest in the use of natural language processing (NLP) techniques, such as machine translation, to automate the ontology translation process in the medical domain has increased [2]. One of the most promising approaches is to use commercial translators that are trained on large-scale text corpora to generate high-quality translations [3]. In a previous study, DeepL proved to be the most accurate translator out of 12 different commercial translators (including Google Translate, Bing, etc.) [4]. With OpenAI’s generative pretrained transformer (GPT) making waves in the technology industry, many experts are excited about its potential to revolutionize NLP [5]. It is therefore a logical step to consider its potential for medical terminology translation. Large language models (LLMs) such as GPT are general-purpose systems capable of performing a broad range of language tasks without explicit task-specific training. This zero-shot capability raises the question of whether LLMs can match or even exceed the translation quality of commercial translation systems – especially in highly specialized domains such as clinical phenotypes, which involve compositional, rare or non-standard terminology.

In this paper, we present a comparative study of terminology translation via GPT-3.5 and the commercial translation software DeepL. Specifically, we focus on the translation of standardized medical terminology contained in the human phenotype ontology (HPO) into German [6]. Importantly, the rare disease community makes extensive use of HPO to perform differential diagnoses. As of January 2024, 11 languages have been integrated into the HPO web interface. These include English, Chinese, Czech, Dutch, Dusun, French, Japanese, Nyangumarta, Spanish, Tiwi, and Turkish [7]. Although there are already initial versions of German translations of the HPO, these translations are incomplete [8].

Translation studies often overlook the range of synonyms for medical terms, which affects their practical use in clinical settings and automated analysis tasks. Many approaches focus on primary terms, resulting in technically correct but incomplete translations. The key value of our study is the comprehensive inclusion of synonyms, which improves accuracy and applicability in medical contexts.

Our study aims to answer the following research questions: How do medical experts rate the quality of the translations in terms of accuracy? To what extent do the translations match reference translations? What is the error level of the generated translations, and how do these errors differ in terms of severity and potential clinical impact?

## Methods

### Study design

We selected 100 random and 20 common terms from the HPO for translation. The 20 common terms were identified by medical professionals from 178 letters from doctors at the Frankfurt Reference Centre for Rare Diseases at the University Hospital Frankfurt. The common terms were selected on the basis of their frequency of use and their significance to the clinical profiles. The selected set of the 120 terms could be assigned to 19 of the 23 top-level categories for phenotypic abnormalities in the HPO, indicating a broad representation across major clinical domains. In addition, the sample included terms of varying ontological depth and specificity, ranging from high-level concepts such as “Abnormal electroretinogram” to highly specific and compositional terms like “Subsarcolemmal accumulations of abnormally shaped mitochondria” or “Absent central microtubular pair morphology of respiratory motile cilia”.

The translations were performed from English to German via GPT-3.5 and DeepL. The synonyms of a term given in the HPO have been included in the translation. A total of 180 synonyms were extracted for the 120 terms. GPT is a LLM that uses deep learning techniques to generate natural language text. It is trained on massive amounts of text data via an unsupervised learning approach, which allows it to learn patterns and relationships in language without the need for explicit annotations. GPT has been shown to be highly effective in a wide range of NLP tasks, including machine translation, text generation, and question answering [9]. DeepL is a commercial machine translation software developed by the German company DeepL GmbH. It is based on neural machine translation techniques, which use deep learning algorithms to learn the statistical patterns of language from large amounts of parallel corpora. The software supports a wide range of languages and domains, including medical terminology, and is optimized for various translation tasks [3].

The exemplary structure of an HPO term can be seen in Table 1. The English version of the HPO is freely available and can be downloaded from the HPO website as an ‘open biomedical ontologies (OBO)’ file [10].

**Table 1:** Structure of the HPO using the term “HP:0000091”. Additional annotations can include definitions, comments, synonyms, cross-references and ontology-typical hierarchical “is_a” dependencies [10]

id: HP:0000091
name: Abnormal renal tubule morphology
def: “An abnormality of the renal tubules.”
comment: The renal tubules are reabsorptive canals that are involved in the secreting, collecting, and conducting of the urine.
synonym: “Abnormality of the renal tubule”
synonym: “Morphologic abnormality of the renal tubules”
xref: UMLS:C4021826
is_a: HP:0012575 ! Abnormal nephron morphology

In GPT-3.5, we created the following prompt for the translation of terms, as it is necessary to specify the intention: *the following terms are used in the Human Phenotype Ontology. Translate them into German. Ensure that the translation has a scientific and medical context.* The terms and synonyms to be translated were subsequently imported into GPT-3.5. GPT-3.5 was used via a web application [11].

The translations made by DeepL in the pre-study are used again in this study as a reference translation for comparison with GPT-3.5 [4]. In the previous study, six medical experts rated the terms. Three medical experts from this study were also involved in the pre-study. The translations generated by DeepL are evaluated again in this study to determine whether the results are consistent with those of the previous study. DeepL was used via the application programming interface (API) [12].

We invited ten medical experts (with medical degrees and several years of clinical or medical research experience) who were fluent in both English and German to evaluate the translations. The experts were blinded to the source of the translations and were not told which translation software was used for each translation, both to reduce the risk of bias. Each translation was rated on a 4-point Likert scale: strongly agree = 1, agree = 2, disagree = 3, strongly disagree = 4. We asked the experts to consider the approval for the respective translation. In addition, medical experts can make comments on individual term or synonym translations.

To answer the question of whether the commercial translators examined are suitable for translating medical terminology, the trend of the experts’ ratings was explored, and an error analysis was conducted. The study was conducted between March 2023 and April 2024.

### Data analysis

We compared the performance of GPT-3.5 and DeepL on the basis of the evaluations provided by medical experts. We calculated the mean and standard deviation (SD) of the Likert ratings (LRs) for each translation software and analyzed the results via statistical tests. The synonyms were always rated together, and the results were then combined with the LR of the main term, resulting in an average LR for each term.

Whether the mean values of the average LR differed significantly between the two systems was measured via the Mann‒Whitney U test. This is a nonparametric statistical test used to compare two independent groups of ordinal scaled variables [13].

Based on the 20 common HPO terms, further research was conducted regarding the quality of the translations. We examined interrater reliability via the intraclass correlation coefficient (ICC) and Fleiss’s kappa. The ICC is used to assess the agreement or reliability of ratings from different raters, with values ranging from 0 (no agreement) to 1 (perfect agreement). The ICC is not a hypothesis test but a measure of consistency of ratings [14]. Fleiss’ kappa also measures the reliability of multiple raters and considers both the observed agreement and the agreement that would be expected by chance, providing a measure of interrater reliability that accounts for the possibility of random agreement. It can take values between − 1 and 1, with 1 indicating perfect agreement, 0 indicating agreement no better than chance, and negative values indicating disagreement beyond chance [15].

To evaluate the quality of the translations, we calculated the Jaro–Winkler similarity between the two translation systems themselves and the similarity to a reference translation of the Health Terminology/Ontology Portal (HeTOP). HeTOP is a comprehensive medical terminology database with translations for diverse medical and clinical applications [16]. Notably, HeTOP does not contain any official German translations for the HPO. For comparison, English HPO terms were searched for in HeTOP and, if available, a German translation was extracted. For the sake of simplicity, the synonyms were not included in this analysis.

The Jaro–Winkler similarity is a string metric that measures the edit distance between two sequences. It uses a prefix scale that rates strings that match from the beginning more favorably. The metric ranges from 0 (indicating no similarity) to 1 (representing identical strings) [17]. Using the Jaro–Winkler similarity metric with a threshold of 0.6, we evaluated the degree of similarity between the machine-generated translations and the HeTOP reference translations. The purpose of this threshold was to focus on significant similarities and ignore instances with low similarity. Any similarity below this value was set to 0.

To assess the impact of term complexity on translation quality, we categorized the 120 HPO terms into four groups based on their length: terms consisting of 1 word, 2–3 words, 4–7 words, and terms longer than 7 words. LRs were calculated separately for each group to evaluate differences in translation accuracy. This stratification also reflects a gradient of ontological depth and specificity, as longer terms often represent more detailed, composite, or rare phenotypic abnormalities. This deliberate heterogeneity was intended to explore how the models handle both broad clinical terms and linguistically complex, granular phenotypes – offering a more nuanced perspective on the model’s semantic understanding and domain adaptability.

## Results

As shown in Table 2, the average LR for the 100 randomly selected HPO terms and 126 synonyms was 1.36 (SD = 0.65) for GPT-3.5 and 1.28 (SD = 0.56) for DeepL. For the 20 common terms and 54 synonyms, the average LR was 1.22 (SD = 0.44) for GPT-3.5 and 1.46 (SD = 0.64) for DeepL. In the pre-study, the LR at DeepL for the same 100 random terms was 1.23, and for the 20 common terms, it was 1.28 [4]. While minor differences in numerical values were observed, the overall pattern and relative ranking of translation quality remained consistent with the pre-study. It can therefore be assumed that the ratings for the translations remain consistent across different experts.

**Table 2:** Average Likert ratings (LRs) and standard deviations (SDs) for the translated HPO terms and synonyms

HPO terms	Pre-Study: DeepL	DeepL	GPT-3.5
100 HPO terms 20 HPO terms	LR = 1.23; SD = 0.50 LR = 1.28; SD = 0.54	LR = 1.28; SD = 0.56 LR = 1.46; SD = 0.64	LR = 1.36; SD = 0.65 LR = 1.22; SD = 0.44

The Mann‒Whitney U test was conducted to assess potential differences in the ratings between DeepL and GPT-3.5, encompassing the 100 randomly selected terms (*p* = 0.27) and the 20 commonly used terms (*p* = 0.06). The null hypothesis for this test states that both groups have the same mean. As the obtained *p* values are not less than the predefined significance level of 0.05, we are unable to reject the null hypothesis. Consequently, there is insufficient evidence to assert that a significant difference exists in the true mean between the two groups. However, it is worth noting that there is a uniformly positive trend in the ratings for both translation tools.

**Fig. 1:**
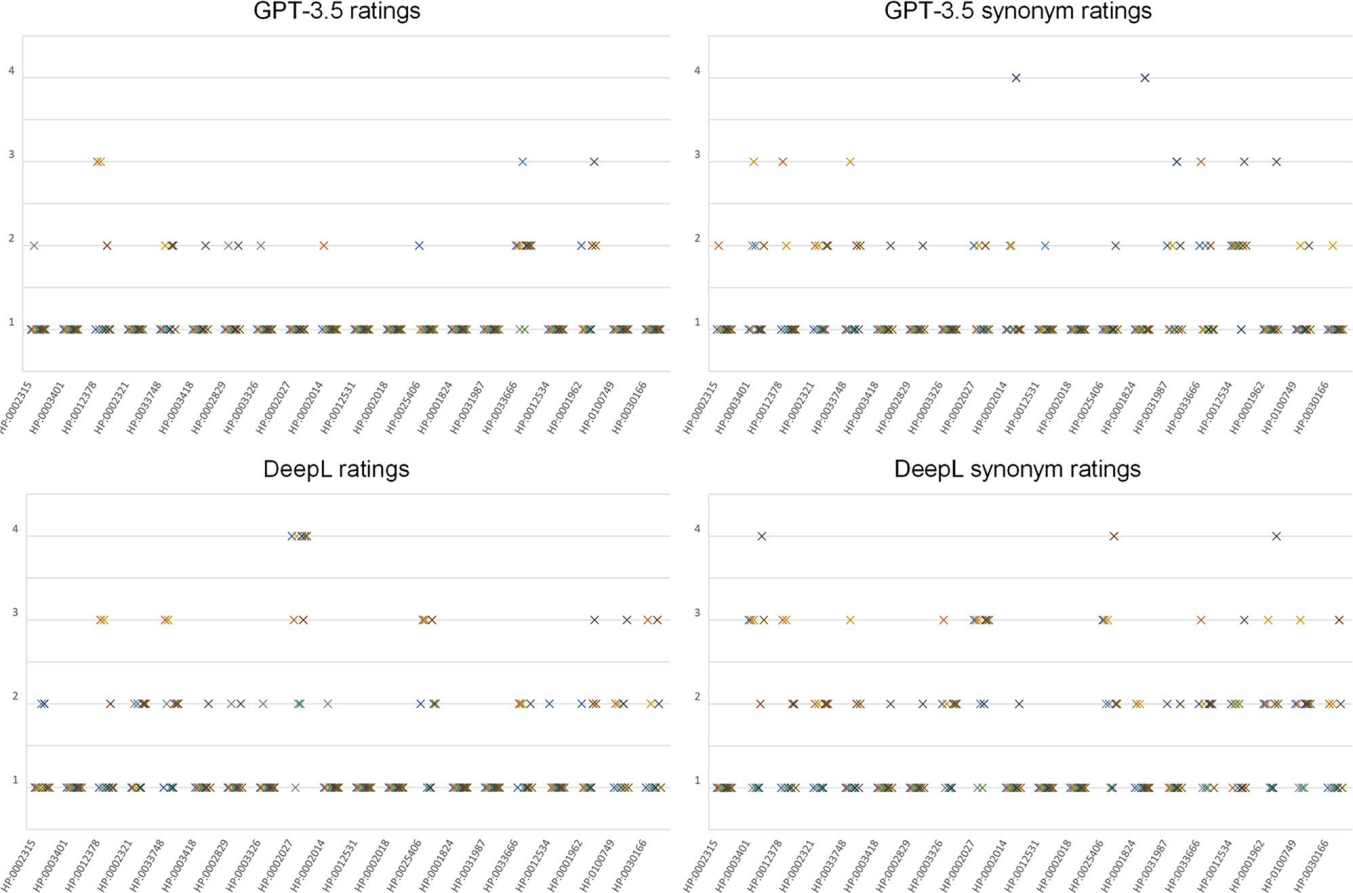
Expert ratings for the 20 translated common HPO terms and their synonyms. The scale ranges from strongly agree (1) with the translation to strongly disagree (4). The color differences indicate the ratings of the different experts

To assess the quality of the translations, we conducted further research focusing on the 20 common terms. Analyzing the ratings assigned to these translations, we observed a consistent pattern of data homogeneity, as depicted in Fig. 1. This homogeneity was manifested by limited variability among the ratings, leading to both the ICC and Fleiss’ kappa yielding low values for all these assessments, as shown in Table 3.

**Table 3:** Average scores for the Fleiss’ kappa coefficient and the intraclass correlation coefficient (ICC) for the ratings of the translations of the 20 common HPO terms by GPT-3.5 and DeepL. The scores indicate the interrater reliability of the expert ratings

	GPT-3.5	DeepL
ICC	0.17	0.36
Fleiss	0.20	0.17

Among the 20 common terms, 15 (75%) had corresponding reference translations in the HeTOP database. After selecting the threshold, the calculated similarities for ratio comparisons yield values of 0.70 for GPT-3.5 versus HeTOP, 0.76 for DeepL versus HeTOP, and 0.76 for GPT versus DeepL, as shown in Table 4.

**Table 4:** Jaro–Winkler similarities for the 20 common HPO terms. The similarities between the translations of GPT-3.5 and DeepL, as well as the respective comparisons to reference translations from HeTOP, were determined

HPO terms	GPT-3.5	DeepL	HeTOP	GPT/HeTOP	DeepL/HeTOP	GPT/DeepL
Headache	Kopfschmerz	Kopfschmerzen	Kopfschmerz	1	0.97	0.97
Paresthesia	Parästhesie	Parästhesie	Parästhesie	1	1	1
Fatigue	Müdigkeit	Müdigkeit	Ermüdung	0	0	1
Vertigo	Schwindel	Vertigo	Vertigo	0	1	0
Hypoesthesia	Hypoästhesie	Hypoesthesie	Hypästhesie	0.98	0.87	0.91
Back pain	Rückenschmerzen	Rückenschmerzen	Rückenschmerzen	1	1	1
Arthralgia	Arthralgie	Arthralgie	Arthralgie	1	1	1
Myalgia	Myalgie	Myalgie	Myalgie	1	1	1
Abdominal pain	Bauchschmerzen	Unterleibs- schmerzen	Adominal-schmerzen	0.62	0.65	0
Diarrhea	Durchfall	Diarrhöe	Diarrhoe	0	0.95	0
Pain	Schmerz	Schmerz	Schmerzen	0.96	0.96	1
Nausea	Übelkeit	Übelkeit	Nausea	0	0	1
Asthenia	Asthenie	Asthenia	Asthenie	1	0.95	0.95
Weight loss	Gewichtsverlust	Gewichtsverlust	Gewichtsverlust	1	1	1
Diminished ability to concentrate	Verminderte Konzentrations- fähigkeit	Verminderte Konzentrations- fähigkeit	n/a	-	-	1
Diminished physical functioning	Verminderte körperliche Funktion	Eingeschränkte körperliche Leistungsfähigkeit	n/a	-	-	0.66
Dysesthesia	Dysästhesie	Dysästhesie	n/a	-	-	1
Palpitations	Herzklopfen	Herzklopfen	n/a	-	-	1
Chest pain	Brustschmerzen	Schmerzen in der Brust	Brustschmerzen	1		0
Night sweats	Nachtschweiß	Nächtliche Schweißausbrüche	n/a	-	-	0.67
			**Jaro mean**	**0.70**	**0.76**	**0.76**

A noteworthy observation made by several experts pertained to the technicality of translations produced by DeepL. However, this variation between more natural and fluent versus more technical and literal translations occurs alternately in both DeepL and GPT, rather than being consistently associated with one translator. Another observation was that GPT-3.5 occasionally generated fewer synonyms in the German output than in the English input (all synonyms were processed together in a single translation batch). Upon closer examination, this reduction seems to occur when several synonyms lead to the same German translation. Furthermore, when synonyms are available in both singular and plural forms, GPT-3.5 often omits the plural in the translation. The error level of the translations generated by GPT-3.5 and DeepL was very low, as indicated by their average ratings. Both had minor linguistic issues, including occasional spelling inaccuracies.

Among the 120 HPO terms, 20 consisted of a single word (17%), 55 terms consisted of 2–3 words (46%), 36 terms consisted of 4–7 words (30%), and 9 terms contained more than 7 words (8%). Notably, among the 20 common HPO terms, 13 out of the 20 terms consisted of a single word, representing 65% of these terms. LR performance varies only slightly among categories: single words score 1.23 (GPT) and 1.26 (DeepL), 2–3 word terms score 1.30 (GPT) and 1.33 (DeepL), 4–7 word terms score 1.36 (GPT) and 1.23 (DeepL), and terms with more than 7 words score 1.49 (GPT) and 1.29 (DeepL).

## Discussion

The ratings provided by the experts for both GPT-3.5 and DeepL indicated that, on average, there were favorable assessments for translation quality. The ratings remained consistent with those from a pre-study, suggesting consistency in expert evaluations and a common understanding of accurate translations. Although minor linguistic inaccuracies, such as “Hypoesthesie” instead of “Hypoästhesie” or “echinovarus” instead of “equinovarus” in DeepL, manifested on occasion, they pose only a minimal risk of clinical misinterpretation. The medical experts found no errors that significantly changed the clinical meaning. However, the subjectivity of the evaluation and the lack of explicit evaluation criteria, such as completeness, comprehensibility, technicality, or syntactic correctness, pose challenges.

Statistical analysis via the Mann‒Whitney U test revealed that there were no significant differences in the mean ratings between GPT-3.5 and DeepL for both the 100 randomly selected terms and the 20 common terms. This suggests that both machine translators performed comparably in terms of translation quality.

High ratings for terms with more than 7 words, such as “Elevated proportion of CD4-negative, CD8-negative, alpha-beta regulatory T cells” or “Anomalous insertion of papillary muscle directly into anterior mitral leaflet”, show promising results even for more complex terms. DeepL is slightly ahead here. The observation that the multi-word translations exhibit comparable performance to their shorter counterparts may be attributed to the incorporation of additional contextual information. In addition to analyzing the influence of term length on translation quality, an alternative approach could involve a range of medical experts in the selection of terms based on their complexity. However, this approach is not without limitations. It is conceivable that medical experts without expertise in translation may subjectively assess the complexity of terms, which could lead to inconsistencies in the selection process.

In assessing interrater reliability, the study revealed data homogeneity among the ratings for the 20 common terms. This resulted in low values for both the ICC and Fleiss’s kappa, indicating that traditional measures of interrater reliability may not be suitable in such cases of minimal variance and uniform ratings. In addition, our analysis revealed instances where the same expert rated identical German translations produced by both translators differently, indicating some degree of inconsistency in rating assignment (intrarater reliability).

To validate translation quality, an independent reference translation from the HeTOP database was employed for 15 of the 20 common terms. The Jaro–Winkler similarity metric revealed high similarities between the machine-generated translations and the HeTOP reference translations. However, it is essential to acknowledge potential nuances, as the threshold may exclude moderately similar yet semantically relevant translations. In cases where the similarity of the terms only slightly exceeds the predefined threshold, as in the comparison between “Bauchschmerzen” and “Abdominalschmerzen”, with a similarity value of 0.62, it becomes clear that the degree of similarity requires careful examination, since the similarity in this case lies solely at the end of the term. In addition, certain terms showed no similarity according to Jaro–Winkler due to the use of alternate synonyms in the translation, despite the fact that these are semantically equivalent.

There are various similarity metrics for measuring text similarity, including the Levenshtein distance, cosine similarity, and Jaccard similarity. However, Jaro‒Winkler stands out because of its ability to weight the prefix (the beginning of words), which is useful for capturing similarities related to singular/plural differences. This allows for a more precise capture of similarities in words, enhancing the detection of semantic similarities. Metrics such as the BLEU (bilingual evaluation under study), which is used in many translation studies [2], are designed primarily for evaluating machine translations when performing 1-to-1 text comparisons with n-gram decomposition and are not necessarily suitable for direct 1-to-1 string comparisons, such as for our comparison of the 20 common terms with the HeTOP database.

It was challenging for some experts to evaluate the quality of the translated synonyms in comparison to their English counterparts. There was a tendency to evaluate the synonyms in relation to the main term. This intricacy is attributable to the specific study design and could be mitigated through the adoption of a randomized presentation format for the translations under evaluation. Experts have also observed instances where synonyms are inaccurately associated with specific terms in the original version of the HPO. For example, in the case of the term “fractured facial bone”, which was one of the 100 randomly selected terms, an English synonym stored as “bone facial bone” was identified that appeared to be mislabeled and that may be more appropriately labeled “broken facial bone”. Since this made it difficult to evaluate the quality of the translated synonym, the rating for this synonym was removed from the overall rating.

GPT-3.5 has several limitations, such as the risk of generating incorrect or biased translations. Providing additional details and contexts through the prompt in GPT-3.5 could improve the accuracy and quality of the translation, especially in regard to medical terminology information, e.g., providing information that many terms might have their roots in Latin or Greek. However, we acknowledge that the optimization of language models such as GPT-3.5 falls under the domain of prompt engineering and that simply adding more information does not guarantee improved results. One possible approach to improve translation quality is to combine translations from multiple translation engines and select different translation candidates from them. This can even be done on the basis of different input languages and support languages [18].

The generalizability of our results to other languages must be viewed critically. In this study, the focus is clearly on translating terminology into German and investigating how well an automated process performs. For validation purposes, it was important to us that medical experts were fluent in both the source language and the target language. However, DeepL has more than 30 source and target languages and can therefore be used for many languages, including French, Korean and Spanish [3]. The GPT models also include various languages in their training data.

The accelerated development of LLMs has led to the introduction of newer GPT models during and following the course of this study. These models are anticipated to introduce novel innovations and enhancements [9, 19]. To facilitate the transfer of the study findings to the translation performance of the current models, the 20 common terms were retranslated with the GPT-4o model. This resulted in translations that were identical except for a few instances of singular/plural differences and minor adjustments to the translations of “Diminished physical functioning” and “Hypoesthesia.” The Jaro–Winkler similarity between GPT-3.5 and GPT-4o was 0.99, whereas the similarity between DeepL and GPT-4o was identical to the similarity between DeepL and GPT-3.5, which was 0.76. These values indicate comparable results, thereby demonstrating uniform validity.

Notably, our study revealed that only 75% of the most common HPO terms had German reference translations in the HeTOP database. Given the limited sample size, the results are not statistically significant. However, given the paucity of studies on extensive translations, these findings underscore the incomplete coverage of translated medical terminology and highlight the importance of our study, particularly for the documentation and diagnosis of rare diseases where precise distinctions in disease characteristics are vital.

## Conclusions

Despite the modest number of evaluated terms in this study, which precludes statistical generalization across the entire HPO, which currently comprises approximately 18,000 terms, the focus of our analysis was on exploring the qualitative capabilities and limitations of GPT and DeepL when confronted with medical terminology. This type of insight is particularly valuable in the current phase of technological integration, where general-purpose artificial intelligence systems are increasingly considered for specialized tasks. GPT and DeepL provide very good translations in the eyes of medical experts. GPT has significant challenges, including unpredictable ambiguities in output, such as sporadic synonym reduction. The presence of arbitrariness and inconsistency in translation, especially within medical terminology, could be a non-negligible problem in clinical practice and automated analysis.

As language models continue to evolve, the choice between machine translators should be made with consideration of their respective strengths. For the initial translation of extensive terminologies such as the HPO, translators such as DeepL show great promise but require additional manual annotation and validation by medical experts. However, the information gain that could be obtained by translating these extensive terminologies, especially for the purpose of describing more complex cases such as rare diseases, should not be neglected.

The complete translation of the HPO into German with DeepL is provided by the corresponding author as an ‘OBO’ file in a repository. In addition, an interactive script was created to perform search queries in the German HPO.

## Data Availability

The complete translation of HPO (v2024–01–16) with DeepL can be found at https://github.com/RichardNoll/HPO_German. The German terms, definitions, and synonyms for a list of HPO codes can also be output via an attached script in Python. It is also possible to identify corresponding HPO codes by entering terms and synonyms in German. The script can be run interactively via a Jupyter notebook in Google Colab.

## Abbreviations

API: Application programming interface
GPT: Generative pretrained transformer
HeTOP: Health terminology/ontology portal
HPO: Human phenotype ontology
ICC: Intraclass correlation coefficient
LLM: Large language model
LR: Likert rating
NLP: Natural language processing
OBO: Open biomedical ontologies
SD: Standard deviation
UMLS: Unified medical language system
